# Functional Reorganization of Local Circuit Connectivity in Superficial Spinal Dorsal Horn with Neuropathic Pain States

**DOI:** 10.1523/ENEURO.0272-19.2019

**Published:** 2019-10-04

**Authors:** Nian Gong, Garo Hagopian, Todd C. Holmes, Z. David Luo, Xiangmin Xu

**Affiliations:** 1 Department of Anesthesiology & Perioperative Care, School of Medicine, University of California, Irvine, Orange, California 92868; 2 Department of Anatomy & Neurobiology, School of Medicine, University of California, Irvine, Irvine, California 92697; 3 Department of Physiology & Biophysics, School of Medicine, University of California, Irvine, Irvine, California 92697

**Keywords:** electrophysiology, excitatory, inhibitory, photostimulation, synaptic connections

## Abstract

The spinal dorsal horn is the first relay structure coding for pain transmission and modulation. Previous anatomical and electrophysiological studies have examined spinal dorsal horn circuit connections and network activity. Further work is required to understand spinal cord sensory information processing that underlies pathological neuropathic pain states. Our previous studies suggest that peripheral nerve injury enhances presynaptic excitatory input onto spinal superficial dorsal horn neurons, which in turn contributes to pathologic nociception. The potential changes in local postsynaptic circuits in the dorsal horn that lead to pathologically heightened behavioral responses to pain remain largely unexplored. We combined whole-cell electrophysiological recordings with laser-scanning photostimulation to test whether peripheral nerve injury in the spinal nerve ligation (SNL) mouse model of neuropathic pain leads to alterations in the functional connectivity of spinal cord circuits including lamina II excitatory interneurons. Here we show that SNL enhances excitation and decreases inhibition to lamina II excitatory interneurons along with their increased glutamate-evoked excitability. The enhanced excitatory postsynaptic input and connectivity evoked by SNL eventually return to normal levels concurrently with the resolution of the neuropathic pain states. The physiological pattern highly correlates with mouse pain behaviors following SNL, supporting a neurophysiological mechanism of central sensitization and neuropathic pain that is functionally localized to the spinal dorsal horn. Together, these data support that SNL induces functional changes in synaptic input and connectivity to lamina II excitatory interneurons that code for pain perception, and thus provide new insights into the mechanism and locus of pain hypersensitivity.

## Significance Statement

Neuropathic pain presumably results from alterations in neuronal circuits that process nociception. This form of pain is often maladaptive. The contribution of circuit connections and detailed local spinal cord circuits underlying neuropathic pain are not well understood. Here, we apply laser-scanning photostimulation combined with whole-cell recordings to investigate local circuit connectivity onto the lamina II interneurons during and after recovery following spinal nerve ligation that causes pathologic neuropathic pain. The present study sheds light on local circuit organization in spinal dorsal horn and shows that reciprocal changes occur in local excitatory interneurons both at the peak and after the gradual normalization of neuropathic pain. This elucidates nociceptive processing changes during and after neuropathic pain conditions and suggests new treatments.

## Introduction

The spinal cord is a major sensory information-processing hub between the peripheral nervous system and the CNS (Willis and Coggeshall, 2004; Todd, 2010; Gong et al., 2017; Gatto et al., 2019). The spinal cord dorsal horn is organized by the pattern of central projections of functionally distinct primary afferents in different laminae (Rexed, 1952; Light, 1992). Lamina II of the dorsal horn, also called substantia gelatinosa, is the major spinal relay area for small-diameter (Aδ and C-fiber) primary afferents carrying noxious, thermal, itch, and innocuous tactile information (Todd, 2010; Braz et al., 2014). Lamina II neurons are defined as interneurons since virtually all cells have axons that remain within the spinal cord (Willis and Coggeshall, 2004). Lamina II interneurons are mostly excitatory (glutamatergic, >70%), but a significant subset of interneurons are inhibitory (GABAergic, <30%). Both subsets receive synaptic inputs from higher brain centers and other local interneurons as well as from the periphery (Todd and Sullivan, 1990; Polgár et al., 2003, 2013; Todd et al., 2003; Maxwell et al., 2007; Todd, 2010). Therefore, lamina II interneurons and their intrinsic circuitry play a crucial role in the maintenance of sensory function by setting the overall “excitability level” and, thus, the output of the superficial dorsal horn neurons (Willis and Coggeshall, 2004). This implies that pathologic pain perception may be modulated in the spinal cord as well as higher brain areas.

Previous anatomic and electrophysiological studies have been performed in an effort to delineate the functional neural circuitry of the superficial dorsal horn (Todd, 2010; Zeilhofer et al., 2012). Lu and coworkers identified an inhibitory pathway in lamina II (Lu and Perl, 2003), an excitatory pathway in lamina I-II (Lu and Perl, 2005), and a normally silent pathway in lamina II-III (Lu et al., 2013). A major limitation of paired recordings in the spinal dorsal horn slices is the low probability of encountering coupled neurons that yields relatively small datasets (often <20 pairs; Graham et al., 2007). More recently, whole-cell patch-clamp recordings combined with photostimulation in slice preparations successfully map the locations of neurons that project locally to an identified single neuron in lamina II of the spinal dorsal horn (Kato et al., 2007, 2009; Kosugi et al., 2013). This method uses light-induced uncaging of caged glutamate to activate presynaptic neurons to map the synaptic input field to morphologically identified single postsynaptic neurons (Callaway and Katz, 1993; Kuhlman et al., 2013; Sun et al., 2016). Here, we apply an improved approach of glutamate uncaging to understand sensory information processing that underlies pain state development following peripheral nerve injury and subsequent recovery processes.

Neuropathic pain is often maladaptive (Todd, 2010). Sensory circuit alterations may involve plastic changes that lead to abnormal pain sensations, such as hyperalgesia (exacerbated pain perception) and allodynia (painful perception to an innocuous stimulus; Cohen and Mao, 2014; Gong et al., 2017). One advance in understanding peripheral nerve injury-induced neuropathic pain is the role of potential changes in the balance of excitation and inhibition in local spinal cord circuits. Pharmacological blockade of GABAergic inhibitory transmission in the spinal cord reveals a normally silent linkage that, when unmasked, facilitates polysynaptic A fiber-mediated excitatory transmission to the recorded lamina II interneurons (Baba et al., 2003) and allodynia (Sivilotti and Woolf, 1994). The potential selective loss of GABAergic interneurons in lamina II spinal dorsal horn may lead to reduced inhibitory tone after spinal nerve injury, causing loss of GABA_A_-mediated IPSCs in lamina II neurons (Moore et al., 2002; Kohno et al., 2003; Scholz et al., 2005; but see Polgár et al., 2005). This disinhibition may lead to an acquired increase in net excitation, which in turn triggers an increased action potential firing rate that may code for neuropathic pain (Zeilhofer et al., 2012). Spinal nerve injury enhances presynaptic excitatory input onto spinal dorsal horn neurons, which contributes to the development of nociception (Zhou and Luo, 2014, 2015). However, dynamic changes in the detailed local postsynaptic circuits in the dorsal horn that drive heightened behavioral pain responses remain largely unexplored.

In the present study, we use laser-scanning photostimulation (LSPS) and whole-cell recordings to characterize and compare local circuit connectivity of the lamina II excitatory interneurons in mouse spinal cord slice preparations under noninjury control conditions, following spinal nerve ligation (SNL) that induces neuropathic pain, and then following functional recovery. Starting with our assessment of detailed maps of excitatory and inhibitory synaptic inputs impinging onto lamina II excitatory interneurons, we show quantitatively how the input strength of excitatory and inhibitory connectivity onto excitatory interneurons in lamina II changes dynamically during the initiation and after the recovery of neuropathic pain states. Our data indicate that the balance of excitatory and inhibitory synaptic connectivity onto lamina II excitatory interneurons is altered following SNL, which leads to neuropathic pain conditions and show that this imbalance resolves coinciding with behavioral recovery from neuropathic pain states.

## Materials and Methods

### Animals

All experiments were performed in accordance with the regulations of the Institutional Animal Care and Use Committee of the University of California, Irvine, and the National Institutes of Health guidelines for animal care and use. All efforts were made to reduce the number of animals used, to minimize their suffering, and to use alternatives to *in vivo* techniques, if available. Male mice (3–15 weeks old) were purchased from Charles River Laboratories for sham and SNL procedures. To genetically label excitatory neurons, vesicular glutamate transporter 2 (VGluT2)-Cre mice (stock #016963, The Jackson Laboratory; Vong et al., 2011) were crossed with Cre reporter Ai9 tdTomato mice (Madisen et al., 2010) from The Jackson Laboratory (stock #007909). The VGluT2-expressing cells were visualized in the offspring VGluT2-Cre; Ai9 mice. All experimental mice were hemizygous for both transgenes (VGluT2-Cre; Ai9). The animals were housed in a room with a 12 h light/dark cycle with *ad libitum* access to food and water.

### Spinal nerve ligation

Unilateral SNL was performed as described previously by Kim and Chung (1992). Briefly, mice (3–4 weeks old) were deeply anesthetized with air-mixed isoflurane (4% for induction, and 1.5% for maintenance). The left L4 spinal nerve, which is anatomically equivalent to the L5 spinal nerve in rat (Rigaud et al., 2008), was exposed and tightly ligated, using a 6.0 silk suture, between the dorsal root ganglion and the junction where spinal nerves form the sciatic nerve. Sham operations were performed as described above, except that the L4 spinal nerve was left intact.

### von Frey filament stimulation

Paw withdrawal thresholds were determined by the up–down method (Dixon, 1980) using a set of von Frey monofilaments (Stoelting). Briefly, each mouse was habituated in a test compartment with a mesh floor for at least 30 min until exploratory behavior had stopped or decreased to a minimal level. The first von Frey filament (0.41 ga) was applied to the plantar surface of the hindpaw until it buckled slightly. If a withdrawal response was observed within 5 s, the next lower weight filament was used. Conversely, if the filament failed to elicit a withdrawal response, the next filament with a higher weight was applied. After the first change in response occurred, this process continued until a total of six responses was recorded, starting from the one preceding the change in response. In the event of four consecutive positive responses to filaments with decreasing weight, or three consecutive negative responses to filaments with increasing weight, a score of 0.01 or 2.0 g, respectively, was assigned. These responses were used to calculate the 50% withdrawal threshold, as described previously (Luo et al., 2001).

### Spinal cord slice preparations

Mice (4–5 weeks old for general recording and 13–15 weeks for recovery recording) were anesthetized with Euthasol (sodium pentobarbital, 100 mg/kg, i.p.) and decapitated. The L4 lumbar region of the spinal cords was removed and cut transversely with a vibratome (VT-1200s, Leica Biosystems) into 400 μm slices in ice-cold, modified, sucrose-containing artificial CSF (SACSF) saturated with 95% O_2_/5% CO_2_ (carbogen). The SACSF contained the following (in mM): 85 NaCl, 75 sucrose, 2.5 KCl, 25 glucose, 1.25 NaH_2_PO_4_, 4 MgCl2, 0.5 CaCl2, and 24 NaHCO3. The slices were first incubated in SACSF, then were transferred to carbogenated ACSF containing the following (in mM): 126 NaCl, 2.5 KCl, 26 NaHCO3, 2 CaCl2, 2 MgCl2, 1.25 NaH_2_PO_4_, and 10 glucose at 32°C for at least 30 min before we began the experimental recordings.

### Slice electrophysiology and laser-scanning photostimulation

Electrophysiological recordings and photostimulation via glutamate uncaging were performed as previously described (Kuhlman et al., 2013). Whole-cell recordings were performed under a differential interference contrast/fluorescent microscope (model BX51WI, Olympus). Oxygenated ACSF at room temperature was perfused into the slice recording chamber through a custom-designed flow system driven by pressurized 95% O_2_–5% CO_2_ (3 psi) at ∼2 ml/min. Slices were examined under a 4× objective for proper targeting of laminae II superficial dorsal horn interneurons that express the red fluorescent protein (RFP), tdTomato. To target whole-cell recordings, cells were visualized at high magniﬁcation (60× objective, 0.9 numerical aperture; LUMPlanFl/IR, Olympus). Cell bodies of recorded neurons were at least 50 μm below the surface of the slice. Patch pipettes (resistance, 4–6 MΩ) made of borosilicate glass were ﬁlled with an internal solution containing the following (in mm): 126 K-gluconate, 4 KCl, 10 HEPES, 4 ATP-Mg, 0.3 GTP-Na, and 10 phosphocreatine, at pH 7.2 and 300 mOsm. In separate recordings in which IPSCs were measured, potassium was replaced with cesium. Electrodes also contained 0.1% biocytin for *post hoc* cell labeling and further morphologic identiﬁcation. Once stable, whole-cell recordings were achieved with good access resistance (usually <30 MΩ), basic electrophysiological properties were examined through hyperpolarizing and depolarizing current injections. Electrophysiological data were acquired with a Multiclamp 700B amplifier (Molecular Devices), data acquisition boards (models PCI MIO 16E-4 and 6713, National Instruments), and a custom-modified version of Ephus software (Suter et al., 2010). Data were digitized at 10 kHz. Any recordings in which the access resistance changed by >20% during the course of the experiment were excluded from the analysis.

Spinal dorsal horn superficial lamina II VGluT2^+^ excitatory neurons were targeted based on RFP expression. The final cell type classification was determined by the combined characterization of RFP expression and electrophysiological properties of the recorded cells. Spinal slices were fixed in 4% paraformaldehyde and transferred to a 30% sucrose solution in PBS. Neurons filled with biocytin during recordings were labeled with Alexa Fluor 488-conjugated streptavidin (1:500 dilution; Jackson ImmunoResearch). Cell morphology and RFP expression were visualized using laser-scanning confocal microscopes (LSM 700 and LSM 780, Carl Zeiss Microscopy).

LSPS was performed under a 4× objective lens. A stock solution of MNI-caged-l-glutamate (Tocris Bioscience) was added to 20 ml of ACSF for a final concentration of 0.2 mm caged glutamate. The spinal slice image, acquired through the 4× objective, was visualized using a high-resolution digital CCD camera, and this image, in turn, was used to guide and register photostimulation sites. Photostimulation (1.5 ms duration, 15–25 mW pulses) from a 350 nm UV laser generator (DPSS Lasers) was delivered to the sample, controlled via an electro-optical modulator and a mechanical shutter. Focal laser spots approximated a Gaussian profile with a diameter of 50–100 μm. Under our experimental conditions, LSPS-evoked action potentials were recorded from stimulation locations within 88 ± 11 µm (*n* = 16) of targeted somata of excitatory neurons and occurred within 150 ms after photostimulation. Together with control experiments, our calibration analysis indicates that LSPS allows for mapping direct synaptic inputs to recorded neurons. Synaptic currents in patched neurons were detected under voltage clamp. By systematically surveying synaptic inputs from hundreds of different sites across a large spinal dorsal horn region, aggregate synaptic input maps were generated for individual neurons. For our mapping experiments, a standard stimulus grid (16 × 16 stimulation sites, 3600 µm^2^ spacing) was used to tessellate the spinal dorsal horn from laminae I to V. The LSPS site spacing was empirically determined to capture the smallest predicted distance in which photostimulation differentially activates adjacent neurons. Glutamate uncaging was delivered sequentially in a no-raster, nonrandom sequence following a “shifting-X” pattern designed to avoid revisiting the vicinity of recently stimulated sites (Shepherd et al., 2003). Because glutamate uncaging agnostically activates both excitatory and inhibitory neurons, we empirically determined the excitatory and inhibitory reversal potentials in spinal dorsal lamina II cells to properly isolate EPSCs and IPSCs. We voltage clamped spinal dorsal lamina II cells at −70 mV to determine LSPS-evoked EPSCs. The holding potential (0–5 mV) was used for IPSC detection with the cesium-containing internal solution.

Photostimulation data analyses were performed as described previously in detail (Shi et al., 2010; Sun et al., 2014). Two major forms of excitatory responses can be induced by photostimulation, as follows: (1) direct glutamate uncaging responses from direct activation of the glutamate receptors of the recorded neuron within the 7 ms window from laser onset; and (2) synaptically mediated responses (EPSCs/IPSCs) resulting from the suprathreshold activation of presynaptic excitatory neurons. To exclude the direct response, candidate EPSCs with arrival times occurring within the direct response window were dismissed. For individual map construction, input measurements from different stimulation sites were assigned to their corresponding anatomic locations in the spinal dorsal horn; color-coded maps of average input amplitude and the number of events per site was plotted to illustrate the overall input pattern to the recorded cells. The input amplitude/strength of each stimulation site was measured by the sum of individual EPSCs or IPSCs from each photostimulation site with the baseline spontaneous response subtracted, and then normalized by the analysis window of 150 ms after photostimulation. This average integrated value was expressed in picoamperes for the analysis window. To quantitatively compare input strength and connections, we measured the total EPSC/IPSC inputs and the numbers of EPSCs/IPSCs across specific spinal dorsal horn subfields for individual cells.

For certain sets of experiments (i.e., VGluT2-expressing neurons and the “putative excitatory neurons” from wild-type mice 1–2 weeks after SNL), the mapping results were qualitatively similar, but there are substantial quantitative differences of the glutamate uncaging results (photostimulation-evoked EPSC sites and average EPSC input strength) between the VGluT2-expressing neurons and the putative excitatory neurons. This could be due to differences in technical parameters for these two different sets of experiments. One possibility is that wild-type and VGluT2 mouse experiments were performed at two different photostimulation and electrophysiology rigs, which had differing uncaging laser power at several months apart. While the mapping precision of LSPS was maintained, VGluT2 mouse experiments had a higher laser power than WT mouse slice mapping. Another possibility is that the recording access resistance differed between these two cohorts of mice. While the results are qualitatively consistent, they are not quantitatively comparable.

### Immunohistochemistry

After physiologic assays, spinal slices were fixed in 4% paraformaldehyde overnight and then transferred to a 30% sucrose solution in PBS. Slices were stained against biocytin with 1:500 Alexa Fluor 488-conjugated streptavidin (Jackson ImmunoResearch) to visualize the morphology of the recorded cells. Slices were also stained for DAPI (Sigma-Aldrich) to identify laminar boundaries.

### Statistical analysis

Data are expressed as the mean ± SEM. For statistical comparisons between two independent groups, normally distributed data were analyzed using the Student’s *t* test. When data were not normally distributed, a Mann–Whitney *U* test was used. For statistical comparisons across more than two groups, we used the Sidak’s multiple-comparisons test (nonparametric one-way ANOVA) and the Mann–Whitney *U* test for group comparisons. Probability values were two tailed, and the statistical significance criterion *p* value was 0.05.

## Results

### LSPS mapping of local synaptic inputs to dorsal horn neurons

**Figure 1.**
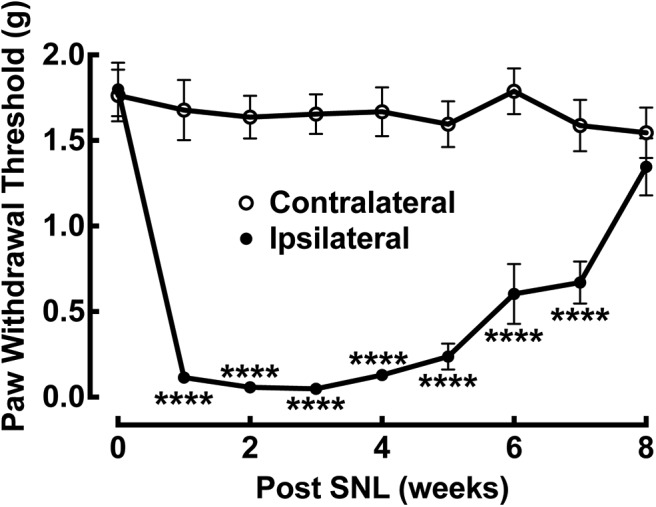
Time course of tactile allodynia development after unilateral L4 SNL in mice (∼4 weeks old). This injury caused a time-dependent reduction in paw withdrawal thresholds on the ipsilateral injury side when compared with the contralateral noninjury side. The hypersensitivity fully developed in 1 week and recovered gradually after 6 weeks of postligation injury. Data presented are the mean ± SEM of 12 mice. ****p < 0.0001 compared with the contralateral side by Sidak’s multiple-comparisons test. Based on this tactile allodynia profile, electrophysiological recordings and circuit mapping were performed on spinal cord slices at two postinjury time points (1–2 weeks and ≥10 weeks postinjury), marking peak neuropathic pain states and subsequent recovery postligation injury, using different cohorts of mice.

Surgical ligation at the L4 spinal nerve in mice (∼4 weeks old) generates neuropathic pain, as measured by increased sensitivity to thermal or mechanical stimuli (Kim and Chung, 1992; Rigaud et al., 2008). This experimental manipulation mimics the major features of clinical neuropathic pain. As shown in Figure 1, unilateral L4 spinal nerve-ligated mice developed tactile allodynia within 1 week after the surgery, as indicated by reduced paw withdrawal thresholds to mechanical stimulation in the injury (ipsilateral) side. Tactile allodynia was not observed in the noninjury (contralateral) side. This behavioral hypersensitivity lasted for at least 6 weeks followed by a gradual recovery.

**Figure 2.**
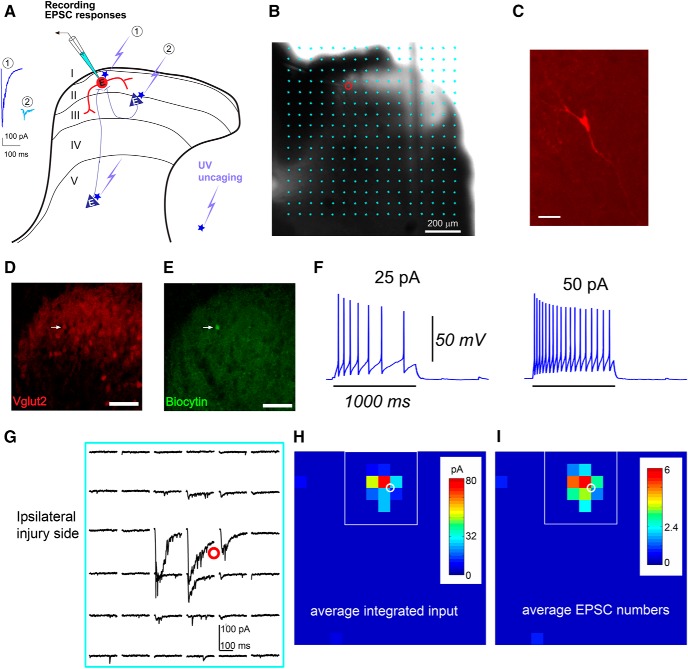
Whole-cell recordings combined with LSPS to map local synaptic inputs to individually recorded lamina II excitatory neuron in L4 spinal cord slices. ***A***, Schematic showing that LSPS maps the broad spatial pattern of synaptic inputs to the neuron of interest in lamina II. Based on response amplitude and latency, we can distinguish direct uncaging responses (1) at perisomatic locations, and synaptically mediated responses (2) to assess circuit inputs from presynaptic neuronal spiking. ***B***, LSPS allows for extensive and quantitative analysis of synaptic inputs to recorded cells from laminar circuits in the whole spinal dorsal horn. The slice image is superimposed with photostimulation sites (cyan circles) spaced at 60 × 60 μm. The red circle indicates the tip of a recording electrode and the cell body location of a recorded neuron. Scale bar, 200 μm. ***C***, The morphology of the recorded neuron is revealed *post hoc* by intracellular biocytin staining (red). Scale bar, 50 μm. ***D***, The confocal image shows red fluorescence of VGluT2 cell bodies in a postfixed VGluT2-Cre:Ai9 mouse slice. ***E***, The recorded neuron is also identified *post hoc* based on its intracellular biocytin staining (green). The arrow indicates the recorded VGluT2-expressing neuron. ***F***, Example of the intrinsic electrophysiological responses of a recorded VGluT2-expressing neuron to intrasomatic current injections from the ipsilateral side of the spinal cord slice. The resting membrane potential is −56 mV. ***G–I***, Example plot of EPSC responses to photostimulation via glutamate uncaging from the recorded neuron (red circle) in the ipsilateral injury side of a spinal dorsal horn slice, within the region shown by the white rectangle in ***H*** and ***I***. The response traces are plotted for 200 ms beginning at the onset of photostimulation. ***G–I***, The raw data shown in ***G*** are quantified and used for the construction of color-coded quantitative input maps (***H***, ***I***). The color scale indicates the average integrated input strength (***H***) and average EPSC numbers (***I***) at individual map sites. The warmer colors indicate stronger input strength (***H***) and larger EPSC numbers (***I***), respectively. The white circle indicates the cell body location of the recorded neuron. Each map site (color pixel) is spaced at 60 × 60 μm.

To map the local spinal cord circuits that code for pain hypersensitivity and to identify the circuit-level changes that underlie chronic pain, we used an LSPS approach combined with whole-cell patch-clamp recordings (Fig. 2*A*). This approach has been widely applied for analyzing cortical circuits (Shepherd et al., 2003; Xu and Callaway, 2009; Xu et al., 2010, 2016a,b; Kuhlman et al., 2013; Sun et al., 2014). Physiologic mapping experiments were performed on both the noninjury (contralateral) and injury (ipsilateral) sides of the spinal cord slices to compare neural circuit connectivity. We measured the connectivity strength and distribution of presynaptic excitatory inputs onto lamina II interneurons in the transverse L4 lumbar region of spinal cord slices. We map the broad spatial pattern of synaptic inputs to recorded lamina II excitatory interneurons at 1–2 weeks after L4 spinal nerve ligation. We also mapped local circuit connectivity in the noninjury contralateral side. Note that this study is technically challenging as we need to overcome technical issues to perform mapping experiments of mature spinal cord circuits in mice (4–5 weeks old for general recording and 13–15 weeks old for recovery recording).

The LSPS approach involves first recording from a single lamina II interneuron, then sequentially stimulating at hundreds of potential presynaptic sites within the LSPS spatial grid (Fig. 2*A*,*B*) via UV uncaging of caged glutamate. Glutamate uncaging generates action potentials from potential presynaptic neurons in uncaging sites, and postsynaptic neuronal recordings provide a quantitative measurement of the spatial distribution and strength of presynaptic excitatory (EPSCs, downward deflecting by convention) or inhibitory (IPSCs, upward deflecting by convention) inputs from lamina I–V spinal dorsal horn to the recorded lamina II interneuron. With subsequent anatomic characterization of the postsynaptic neurons (local excitatory interneurons; Fig. 2*C–E*), as well as its intrinsic electrophysiological firing properties (Fig. 2*F*), the cell type can be classified, allowing a map of input sources to be generated for an identified cell type. Further, subsequent photostimulation data analysis can be performed, which distinguishes direct uncaging responses and synaptic input responses of recorded neurons (Fig. 2*A*,*G–I*).

**Figure 3.**
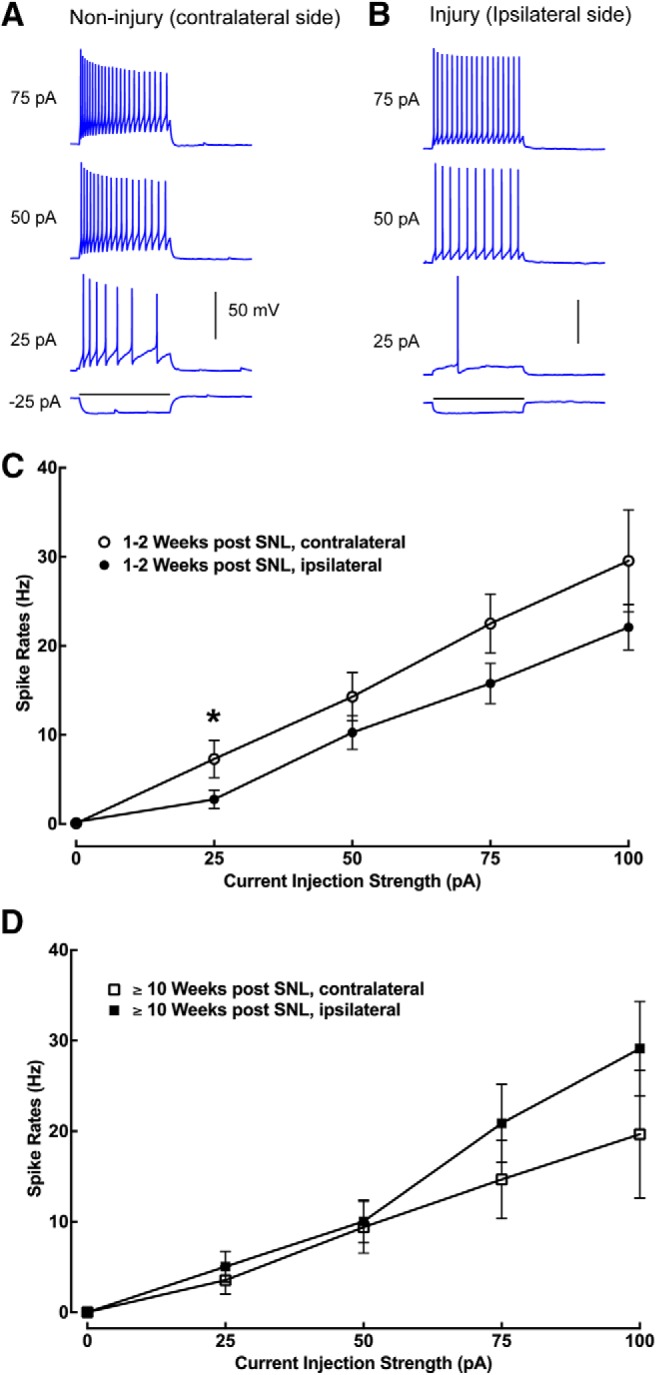
Intrinsic membrane excitability of recorded neurons does not show major changes under SNL conditions or after recovery. ***A***, ***B***, Intrasomatic current injection evoked response traces of example lamina II excitatory neurons recorded from the noninjury (contralateral) side and the injury (ipsilateral) side of spinal cord slices, respectively. ***C***, The relationship between spiking rates and current injection strengths of recorded neurons from the contralateral (*n* = 11–12 cells) and ipsilateral (*n* = 18–19 cells) side of mouse L4 superficial dorsal horn during the initial response to injury between 1 and 2 weeks following injury. Except for the difference at the current injection of 25 pA (*p = 0.041), there is no significant difference between the plotted data points at other current injection strengths. ***D***, The relationship between spiking rates and current injection strengths of recorded neurons from the contralateral (*n* = 6–10) and ipsilateral (*n* = 8–10) side of mouse L4 superficial dorsal horn at ≥10 weeks following injury. Data are presented as the mean ± SEM.

Excitatory interneurons are genetically labeled through a Cre-directed approach by crossing the VGluT2-Cre mouse (Vong et al., 2011) to the Ai9 tdTomato reporter mouse (Madisen et al., 2010). Recordings of excitatory interneurons are facilitated by the identification of red fluorescence in the tdTomato expressing VGluT2-Cre; Ai9 mice (Fig. 2*D*,*E*). These excitatory cells display intrinsic properties of regular firing patterns (Figs. 2*F*, 3*A*,*B*), largely consistent with previous findings in rats (Maxwell et al., 2007; Yasaka et al., 2010). However, a potential caveat is noted that the tonic firing can also often be seen in inhibitory interneurons in rat superficial dorsal horn (Yasaka et al., 2010). Under our experimental conditions, photostimulation-evoked neuronal excitability profiles indicated that LSPS has sufficient spatial resolution for spinal lamina II circuit mapping (see below).

### Enhanced local excitation and decreased inhibition to lamina II excitatory interneurons contribute to neuropathic pain states

Since the intrinsic excitability of lamina II neurons could potentially influence the induction of plasticity, we investigated the intrinsic electrophysiology of the recorded excitatory interneurons under neuropathic pain conditions (1–2 weeks) and after recovery (≥10 weeks). These excitatory neurons exhibited relatively uniform and linear properties of spiking frequency, adaptation, and spike shapes in response to intrasomatic current injection. While recorded neurons in the noninjury (contralateral) side of the spinal cord slices show a higher spiking rate than those recorded in the injury (ipsilateral) side of slices (*p* = 0.04) within 1–2 weeks post-SNL, there was no significant difference in evoked spike frequency between the groups of recorded neurons over other current injection strengths (Fig. 3). This shows that the intrinsic membrane excitability of recorded neurons does not show dramatic changes during the initial response to injury between 1 and 2 weeks following injury, which coincides with neuropathic pain conditions, or at 10 weeks following injury, which is marked by functional recovery from pain states. Thus, intrinsic neuronal properties in response to injury do not largely account for postinjury neuropathic pain or recovery.

**Figure 4.**
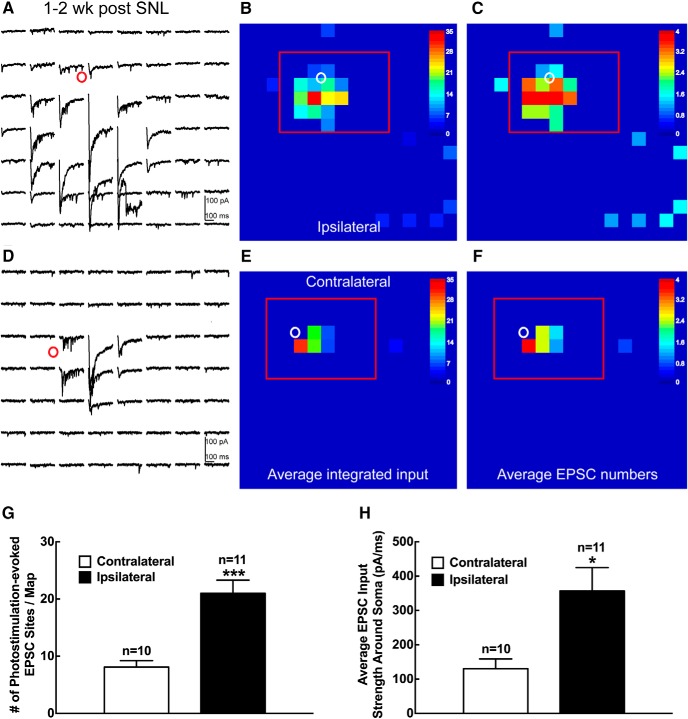
Enhanced local excitatory synaptic circuit connections to superficial dorsal horn VGluT2-expressing excitatory neurons 1–2 weeks after SNL in mouse L4 spinal cord slices. ***A–F***, Example plot of EPSC responses to photostimulation via glutamate uncaging from the selected excitatory neuron (red circle) on the ipsilateral (***A***) or contralateral (***D***) side within the region shown by the red rectangle in ***B*** and ***C***, and ***E*** and ***F***, respectively. The response traces are plotted for 200 ms beginning at the onset of photostimulation. The raw data shown in ***A*** and ***D*** are quantified and used for the construction of color-coded quantitative input maps (***B***, ***C*** and ***E***, ***F***, respectively). The color scale indicates the average integrated input strength (***B***, ***E***) and average EPSC numbers (***C***, ***F***) at individual map sites. The warmer colors indicate stronger input strength (***B***, ***E***) and increased EPSC numbers (***C***, ***F***), respectively. The white circle indicates the cell body location of the excitatory neuron. Each map site (color pixel) is spaced at 60 × 60 μm. ***G***, ***H***, Summary data showing the number of photostimulation-evoked EPSC sites per map (***G***) and the average EPSC input strength around the soma (***H***) measured from excitatory neurons on the ipsilateral (*n* = 11) and contralateral (*n* = 10) sides. Data are presented as the mean ± SEM. *p < 0.05 and ***p < 0.001 compared with the contralateral side by Student’s *t* test.

**Figure 5.**
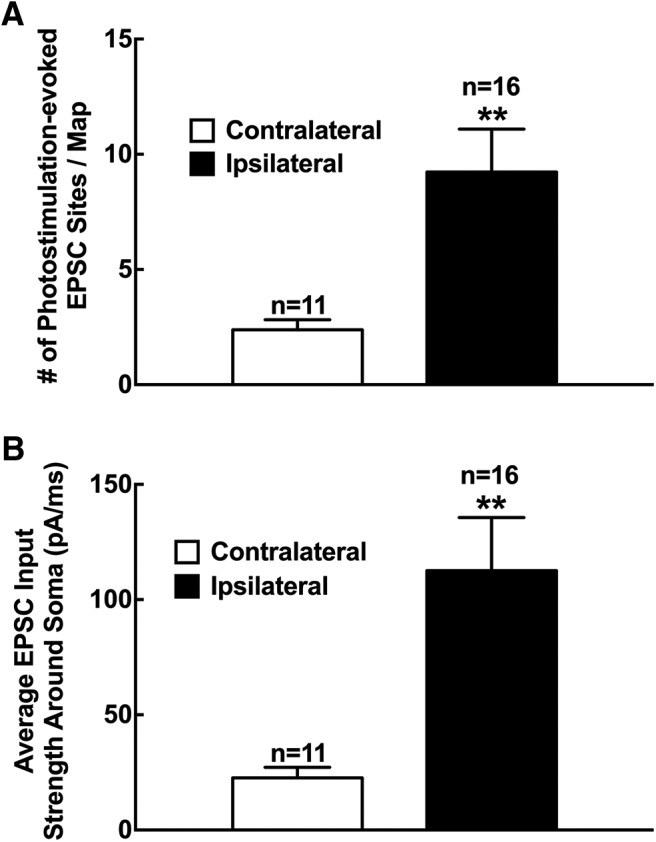
Increased local excitatory synaptic circuit connections to putative excitatory neurons in superficial dorsal horn at 1–2 weeks after SNL in mouse L4 spinal cord slices. ***A***, ***B***, Summary data showing the number of photostimulation-evoked EPSC sites per map (***A***) and the average EPSC input strength around the soma (***B***) measured from putative excitatory neurons on the ipsilateral (*n* = 11) and contralateral (*n* = 16) sides. Data are presented as the mean ± SEM. **p < 0.01 compared with the contralateral side by Student’s *t* test.

**Figure 6.**
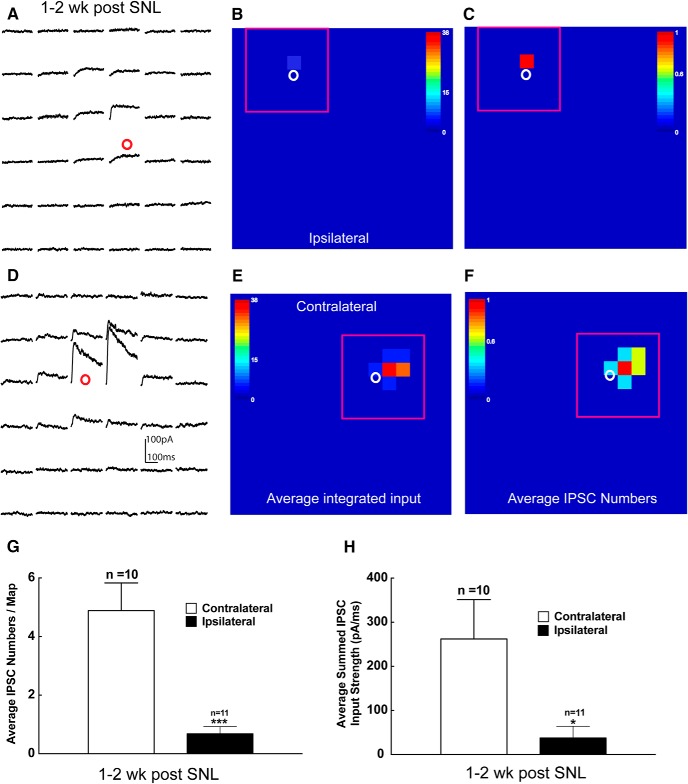
Decreased local inhibitory synaptic circuit connections to superficial dorsal horn excitatory neurons 1–2 weeks after SNL in mouse L4 spinal cord slices. ***A–F***, Example plot of IPSC responses in response to photostimulation from the selected excitatory neuron (red circle) on the ipsilateral (***A***) or contralateral (***D***) side within the region shown by the red rectangle in ***B*** and ***C***, and ***E*** and ***F***, respectively. The response traces are plotted for 200 ms beginning at the onset of photostimulation. The raw data shown in ***A*** and ***D*** are quantified and used for the construction of color-coded quantitative input maps (***B***, ***C*** and ***E***, ***F***, respectively). The color scale indicates the average summed IPSC input strength (***B***, ***E***) and average IPSC numbers (***C***, ***F***) at individual map sites. The warmer colors indicate stronger input strength (***B***, ***E***) and increased IPSC numbers (***C***, ***F***), respectively. The white circle indicates the cell body location of the excitatory neuron. Each map site (color pixel) is spaced at 60 × 60 μm. ***G***, ***H***, Summary data showing the number of photostimulation-evoked IPSC sites per map (***G***) and the average summed IPSC input strength around the soma (***H***) measured from excitatory neurons on the ipsilateral (*n* = 11) and contralateral (*n* = 10) sides. Data are presented as the mean ± SEM. *p < 0.05 and ***p < 0.001 compared with the contralateral side by Student’s *t* test.

LSPS mapping reveals a striking difference in the synaptic inputs to recorded lamina II excitatory interneurons 1–2 weeks following spinal nerve ligation (Figs. 4–6). When compared with the control noninjured contralateral side, excitatory neurons from the injured ipsilateral side have much stronger and more intensive excitatory inputs, indicated by both the average integrated input amplitude and EPSC event frequency. Specifically, we find that under neuropathic pain conditions at 1–2 weeks following SNL injury, lamina II excitatory interneurons on the ipsilateral side receive stronger and more extensive local excitatory synaptic inputs and also have more direct uncaging responses compared with the noninjured contralateral side (Fig. 4*A*,*D*). The strength of integrated input (Fig. 4*B*,*E*) and the numbers of EPSC events (Fig. 4*C*,*F*) within the analysis window reveal that the frequency and strength of excitatory input received by the recorded excitatory neurons on the ipsilateral side increase by 2.6 (21.0 ± 2.3 vs 8.1 ± 1.1 events) and 2.7 (356.7 ± 68.1 vs 130.3 ± 28.6 pA) times that of the control contralateral side, respectively (Fig. 4*G*,*H*). These findings are replicated by our recordings from putative excitatory interneurons in lamina II of spinal dorsal horn in a cohort of wild-type mice at 1–2 weeks following SNL injury (Fig. 5). We determined putative excitatory neurons by visualizing their relatively uniform morphology in live slices and their action potential firing characteristics guided by our recorded data from VGluT2-expressing cells (Figs. 2, 3). Note that while enhanced local excitatory synaptic circuit connections to superficial dorsal horn VGluT2-expressing excitatory neurons and putative excitatory neurons are qualitatively similar, there are substantial quantitative differences of the glutamate uncaging results (photostimulation-evoked EPSC sites and average EPSC input strength) between the VGluT2-expressing neurons and the putative excitatory neurons from wild-type mice. This is likely due to the potential effects of differences in laser power and recording access resistance difference between the cohorts (see Materials and Methods). Based on these factors, we report the results as being qualitative in nature rather than being quantitatively comparable.

Disinhibition of spinal excitatory interneurons is an important mechanism underlying neuropathic pain (Scholz et al., 2005; Meisner et al., 2010; Lu et al., 2013; Todd, 2015). This may contribute to the increased number of excitatory inputs received by the recorded lamina II excitatory interneurons following injury. We used the LSPS strategy to directly map inhibitory inputs to the recorded excitatory neurons. Inhibitory inputs are distinguished by their holding properties at different membrane potentials (for details, see Materials and Methods). As shown in Figure 6, recorded lamina II excitatory interneurons on the ipsilateral injured side display weak and sparse inhibitory circuit connections (Fig. 6*A–C*). In contrast, local inhibitory connections to the recorded lamina II excitatory interneuron on the contralateral noninjured control side are strong and extensive (Fig. 6*D–F*). The average IPSC event frequency and integrated input amplitude on the ipsilateral injured side decreased by 85.7% (0.7 ± 0.2 vs 4.9 ± 0.9 events) and 85.6% (37.7 ± 26.3 vs 262.0 ± 89.1 pA) compared with the contralateral control side at 1–2 weeks post-SNL injury, respectively (Fig. 6*G*,*H*). Thus, superficial dorsal horn hyperexcitability following injury is due to changes in both excitatory and inhibitory input integration in lamina II excitatory interneurons. The imbalance of stronger excitatory connections and weaker inhibitory connections on the ipsilateral injured side versus the contralateral control side temporally correlates with the behavioral neuropathic pain response following injury, as shown in Figure 1.

Please note that our recorded cells received strong excitatory and inhibitory input mainly from positions that were close to their soma, a pattern that is relatively invariant for all excitatory interneurons throughout lamina II. Supporting our finding, Kato et al. (2007) also found that the synaptic inputs to lamina II islet cells arose almost entirely from within lamina II, and no inputs were found from the deep dorsal horn. It remains to be investigated whether other classes of lamina II neurons might preserve such input patterns under neuropathic pain conditions.

**Figure 7.**
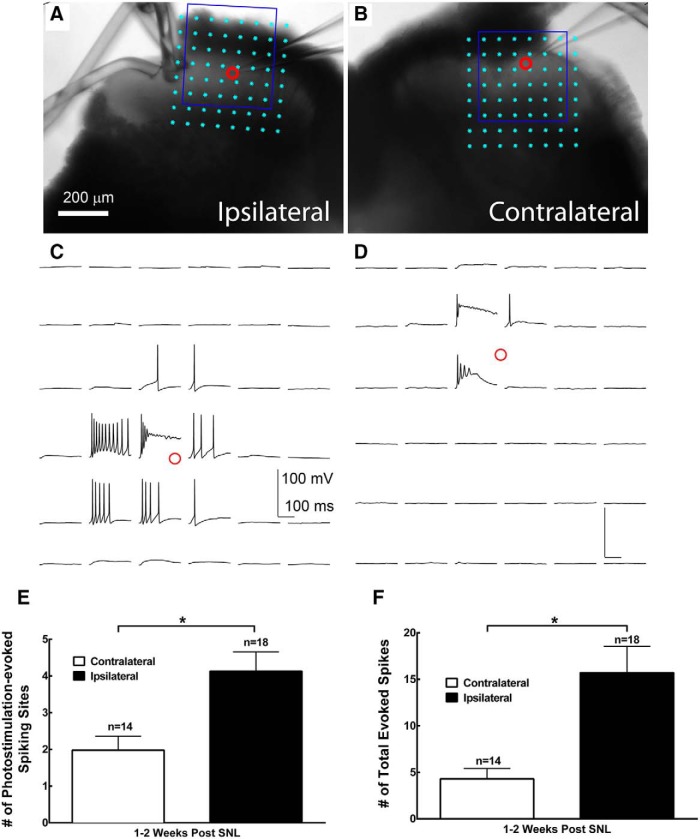
SNL increases glutamate-evoked excitability in superficial dorsal horn putative excitatory neurons 1–2 weeks after injury. ***A–D***, Spiking activities through laser-scanning photostimulation via glutamate uncaging are used to assess the evoked excitability of recorded neurons in L4 spinal cord slices. L4 spinal dorsal horn slice images superimposed with photostimulation sites (cyan circles) are spaced at 60 × 60 μm. ***A***, ***B***, The red circle indicates the tip of a recording electrode and the cell body location of the recorded neurons from the ipsilateral (***A***) and contralateral (***B***) sides of the spinal cord. The plots of photostimulation responses from the recorded neurons within the blue square in ***A*** and ***B*** are shown in ***C*** and ***D***, respectively. Current-clamp recording was used to examine suprathreshold spiking activities in recorded neurons. ***E***, ***F***, Plots of photostimulation-evoked spiking sites (***E***) and total evoked spike number (***F***) per map for neurons recorded from the contralateral (*n* = 14) and ipsilateral (*n* = 18) side of the spinal cord. Data are presented as the mean ± SEM. *p < 0.05 compared with the contralateral side by Student’s *t* test.

A component of the imbalanced excitatory and inhibitory inputs to ipsilateral lamina II excitatory interneurons following injury is due to stronger glutamate-evoked excitability of the recorded putative excitatory interneurons on the ipsilateral side relative to the contralateral control side, as shown in Figure 7. We assessed the neuronal excitability profiles of lamina II putative excitatory interneurons from wild-type mice. These neurons were recorded in whole-cell current-clamp mode, and their excitability profiles were assessed by the number of photostimulation-evoked spiking sites and total evoked spikes at 1–2 weeks following SNL injury. Compared with the contralateral control side, the number of photostimulation-evoked spiking sites on the ipsilateral injured side increases by 109.0% (4.1 ± 0.5 vs 2.0 ± 0.4 sites) and the total evoked spikes per map increases by 265.6% (15.7 ± 2.8 vs 4.3 ± 1.1 spikes; Fig. 7*E*,*F*). Since photostimulation-evoked spiking occurred on direct stimulation, the broad changes in evoked hypersensitivity are consistent with the postsynaptic change in spinal dorsal horn neuron hyperexcitability post-SNL. The resulting increase in lamina II neuronal excitability after spinal nerve ligation likely accounts for the enhanced activation following peripheral stimulation, thus contributing to postinjury neuropathic pain states.

### “Normalized” local circuit excitation correlated with behavioral recovery from pain states

**Figure 8.**
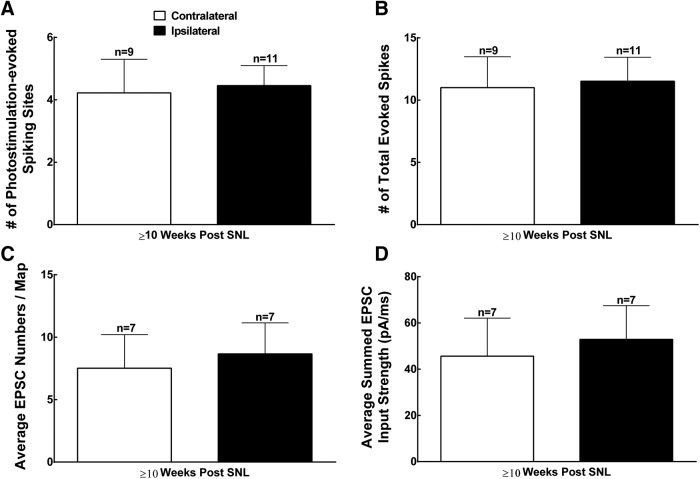
Measurements of local synaptic circuit connections in superficial dorsal horn at ≥10 weeks after SNL. ***A***, ***B***, Plots of photostimulation-evoked spiking sites (***A***) and total evoked spike number (***B***) per map for neurons recorded from the contralateral (*n* = 9) and ipsilateral (*n* = 11) side of the L4 spinal slices. ***C***, ***D***, Plots of photostimulation-evoked EPSC sites per map (***C***) and average EPSC input strength around the soma of recorded neurons (***D***) measured on the ipsilateral (*n* = 7) and contralateral (*n* = 7) sides. There is no significant difference between the recorded neurons on the contralateral and ipsilateral sides of L4 spinal slices.

The analysis of pain behavior in Figure 1 shows a gradual recovery from abnormal pain hypersensitivity to mechanical stimulation that resolves at 8–10 weeks postinjury, which is consistent with previous reports (Kim and Chung, 1992; Hogan et al., 2000; Luo et al., 2001; Li et al., 2004; Rigaud et al., 2008; Guan et al., 2010; Kim et al., 2012). The mechanism of this recovery is still unclear. To test whether normalization of heightened excitability of lamina II excitatory interneurons correlates with the recovery of SNL-induced behavioral hypersensitivity, we examined the glutamate-evoked excitability profiles and excitatory connections to lamina II putative excitatory interneurons in both the ipsilateral and contralateral sides of the spinal cord slices at least 10 weeks after spinal nerve ligation. As shown in Figure 8, there was no significant difference in the recorded putative excitatory interneurons between the ipsilateral and contralateral sides in terms of the number of photostimulation-evoked spiking sites and total evoked spikes, and the average EPSC numbers and integrated excitatory input. Compared with the mapping data from VGluT2-expressing excitatory neurons and putative excitatory neurons at 1–2 weeks after SNL (Figs. 4, 5, 7), lamina II putative excitatory interneurons recorded in the ipsilateral side of the spinal cord slices show a strong tendency of reduced local circuit excitation and decreased glutamate-evoked excitability. Interestingly, a closer look indicates that the cells recorded in the contralateral side of the spinal cord slices seem to have increased their excitability at ≥10 weeks after spinal nerve ligation when compared with those of 1–2 weeks after SNL (Figs. 5, 7). This suggests a possibility that behavioral recovery with long post-SNL intervals can potentially result from both ipsilateral and contralateral circuit connection changes. The quantitative degree of these differences requires further work. Together, the functional changes of local excitatory connections at lamina II indicate that circuit plasticity within this structure likely contributes to both early-stage postinjury neuropathic pain responses and their eventual recovery.

## Discussion

Neuropathic pain following injury and recovery is a complex phenomenon whose neural mechanism is not entirely understood. In this study, we used whole-cell electrophysiological recordings combined with LSPS to quantitatively and spatially map the local excitatory and inhibitory synaptic inputs into lamina II excitatory interneurons at postinjury time points marking peak neuropathic pain states and subsequent recovery. SNL injury does not significantly affect the intrinsic membrane excitability of recorded excitatory lamina II interneurons on either the ipsilateral injured side or the contralateral control side. In contrast to injury-induced changes in intrinsic membrane excitability in DRG neurons following nerve injury (Hong et al., 2004; Kirita et al., 2007; Wang et al., 2007), our data show that the intrinsic membrane excitability of recorded lamina II excitatory interneurons does not show major changes post-SNL. Instead, the glutamate-evoked neuronal excitability on the ipsilateral side is dramatically enhanced following SNL injury.

SNL injury enhances local excitatory synaptic circuitry inputs and decreases local inhibitory synaptic circuitry inputs to excitatory lamina II interneurons. This coincides with peak neuropathic pain states 1–2 weeks following injury. Further evidence that input integration at lamina II excitatory neurons accounts for postinjury neuropathic pain state development is shown by the finding that reduced excitatory postsynaptic connectivity and decreased glutamate-evoked excitability of ipsilateral lamina II excitatory neurons occur when the nerve-injured mice functionally recover from neuropathic pain states (Fig. 1). The superficial dorsal horn circuitry changes strongly correlate with the behavioral sensitivity to stimuli, thus indicating their mechanistic contribution to injury-induced neuropathic pain processing and recovery. Our conclusions are also supported by earlier work *in vivo* (Bourane et al., 2015; Peirs et al., 2015; Saloman et al., 2015; Gatto et al., 2019). A DREADD (designer receptors exclusively activated by designer drugs) approach was used in which hM3Dq receptors were selectively expressed in lamina III of the dorsal horn of VGluT3-Cre mice. DREADD-induced activation of VGluT-containing neurons evoked mechanical hypersensitivity and allodynia (Peirs et al., 2015). The present study addresses neural mechanisms based on *in vitro* circuit-mapping experiments. In the future, we plan to perform genetically targeted manipulation of lamina II excitatory interneurons *in vivo* to reveal detail neurophysiological mechanisms of central sensitization and neuropathic pain post-SNL.

We demonstrate that glutamate-evoked excitability of excitatory interneurons on the injured side coincides with the onset of peak tactile allodynia 1–2 weeks following SNL injury. The increase of glutamate-evoked excitability occurs specifically in neurons recorded on the injury side in contrast to the absence of hyperexcitability responses on the control noninjured side of the superficial dorsal horn. This argues strongly against a nonspecific effect from surgical procedures or tissue harvesting. We consider that SNL-induced sensory pathway hyperexcitability can result from both peripheral and central sensitization, including sensitized dorsal root ganglion neurons (Hogan et al., 2000; Tang et al., 2012) that can modulate primary afferent inputs (Xie et al., 2010; Zhou and Luo, 2014, 2015; Li et al., 2016). Furthermore, interneuron firing rates may increase in response to noxious or innocuous stimuli (Cui et al., 2016; Patel and Dickenson, 2016). Our dynamic mapping of excitatory and inhibitory synaptic circuitry input into lamina II excitatory interneurons supports our hypothesis that overall superficial dorsal horn hyperexcitability changes following SNL injury are at least partially due to both enhanced excitatory synaptic excitation and reduced inhibition onto excitatory interneurons. These results are consistent with a recent study (Kopach et al., 2017) that shows increased activity of superficial dorsal horn excitatory interneurons and reduced excitability of inhibitory interneurons in spinal cord-injured animals that exhibit pain states and spasticity. It remains to be determined whether changes in other classes of lamina II neurons also contribute to neuropathic pain state development following injury.

Normalization of synaptic circuitry input into lamina II excitatory interneurons coincides with the recovery of neuropathic pain states in the SNL model. However, the mechanisms underlying the resolution of neuropathic pain states following nerve injury are still unclear. Recent findings suggest that successful axon regeneration contributes to the functional recovery following nerve injury (Mar et al., 2014; He and Jin, 2016). Injured fibers can measurably regenerate as early as 4 weeks after SNL injury. The regenerating nerve fibers generate abnormal spontaneous activity during the period of heightened behavioral hypersensitivity. Perhaps successful regeneration with correct target reinnervation contributes to the recovery of pain states (Xie et al., 2017) through the normalization of local circuit excitation in the spinal dorsal horn.

In summary, our data show that enhanced neuron excitability and imbalanced excitatory and inhibitory synaptic connectivity to lamina II excitatory interneurons coincides with the peak of behavioral hypersensitivity following injury and that their resolution to control baseline levels coincides with functional recovery from injury-induced tactile allodynia. Together, the results support our hypothesis that SNL induces maladaptive changes in synaptic input and connectivity to lamina II excitatory interneurons in the dorsal horn and provide a new insight for understanding spinal local circuitry modulation/reorganization under neuropathic pain conditions.

## Synthesis

Reviewing Editor: Trevor Drew, University of Montreal Decisions are customarily a result of the Reviewing Editor and the peer reviewers coming together and discussing their recommendations until a consensus is reached. When revisions are invited, a fact-based synthesis statement explaining their decision and outlining what is needed to prepare a revision will be listed below. The following reviewer(s) agreed to reveal their identity: Andrew J. Todd.

The authors have made some of the changes requested by the Reviewers, but I think that there are some important issues that need to be addressed.

(1) There are substantial differences between the glutamate uncaging results from the VGLUT2-expressing neurons (Fig 4) and the “putative excitatory neurons” from wild-type mice (Fig 5). For example, comparing Figs 4G and 5A there seem to be around twice as many photostimulation-evoked EPSC sites, even though the difference between ipsilateral and contralateral sites is maintained. There is also a substantial discrepancy for “Average EPSC input strength around soma” (compare Figs 4H and 5B). This suggests that absolute values cannot be compared between experiments, and needs some explanation.

(2) Following on from this, the apparent “normalising” shown at long post-SNL intervals (Fig 8) could potentially result from a change on the contralateral side. It is difficult to justify the statement on p17-18 that “baseline excitatory postsynaptic input levels . . . resolve to pre-injury control levels”, because the results shown for the 2 sides in Fig 8A-C are very different from the contralateral values shown in Figs 7E,F and 5A.

(3) I am not at all convinced by the apparent targeting of excitatory neurons in the wild-type mice. The firing patterns shown in the VGLUT2 mice (Fig 2F) resemble the tonic firing that is often seen in inhibitory neurons (Yasaka et al 2010), and the level of morphological detail shown in Fig 2C would certainly not allow excitatory neurons to be distinguished from inhibitory ones. Both inhibitory and excitatory interneurons in lamina II are very diverse in terms of dendritic morphology, and it would require excellent labelling to be able to distinguish between them. Essentially the recordings made in wild-type mice could have been from either class of neuron.

Minor points:

line 19: “pain perception”.

line 44: “laminated layers” is a tautology - laminated means having layers.

line 73: insert “the role of” (or similar) before “potential changes”.

lines 277-9: the finding of “regular firing patterns” (presumably tonic firing) in VGLUT2+ cells is not particularly consistent with results of Maxwell et al or Yasaka et al.

line 312: “wild type mice”.

line 313: “We identified putative excitatory neurons by visualizing . . .” (but see point 3 above).

line 326: “decrease by 6.0 fold” is confusing - better to say “decreased to one-sixth” (or percentage).

lines 368-71: presumably “functional recovery” does not contribute to early stage neuropathic pain - just to the eventual recovery.
